# Converge of data science and laboratory medicine

**DOI:** 10.1515/almed-2024-0164

**Published:** 2024-11-21

**Authors:** Javier Nieto-Moragas, Anna Marull Arnall, Fernando Calvo Boyero, Salomón Martin Perez, Fernando Marqués García, Javier Hernando Redondo, Albert Blanco Grau, Cristian Cauqui Lende, Ángel Molina Borrás, Daniel Prieto Arribas, Elena de Rafael González

**Affiliations:** SEQC^ML^ Data Science Working Group; Hospital Universitari de Bellvitge, Hospitalet de Llobregat, Spain; Girona Dr Josep Trueta University Hospital, Girona, Spain; Virgen Macarena University Hospital, Sevilla, Spain; Germans Trias i Pujol Hospital, Badalona, Spain; Hospital del Mar de Barcelona, Barcelona, Spain; Vall d’Hebron University Hospital, Barcelona, Spain; 12 de Octubre University Hospital, Madrid, Spain; Hospital Universitari de Girona Dr Josep Trueta, Girona, Spain; Hospital Clínic de Barcelona, Barcelona, Spain; La Paz University Hospital, Madrid, Spain; Complejo Hospitalario Universitario de Toledo, Toledo, Spain

**Keywords:** machine learning, working group, data science, training

Data science is a discipline that uses knowledge and insights to extract and transform data for analysis and presentation. This process requires prior verification of the quality, robustness and interpretability of data. Clinical laboratory professionals are perfectly familiar with managing this sort of data flows, since computational methods and the achievement of processes, known as pipelines, present certain parallelism to the handling of samples in laboratories. In data analysis, different tools are used to determine data quality, the format is adapted for representation and modelling, and finally the result is provided in a report for interpretation.

The synergy between data science and laboratory medicine has increased in the recent years and has attracted the attention of clinical laboratory professionals. This fact is not surprising, as these professionals work with large sets of data of different nature obtained using distinct analytical methods. Data science tools extract relevant information from subsets of complex data that would be difficult to interpret as individual data. Interest in integrating data science applications in laboratories has been growing in several countries at an increasing pace since the COVID-19 pandemic. Some components of data science have gained special relevance in the recent years, including artificial intelligence, big data, and data mining. The application of these techniques in Medicine has enabled the development of rapid and accurate diagnostic models, improved therapeutic decision-making and enhanced real-time patient monitoring. Data science can be easily integrated into clinical practice, as the latter uses real-time raw data (images, audio or electrical signals) that can be effortlessly interpreted by artificial intelligence or exploited using data mining and big data. Structured data (displayed in tables, lists or worksheets) is widely used retrospectively, along with machine learning algorithms to optimize turnaround times and facilitate data interpretation. These tools are currently grouped into the terms Real World Data (RWD) and Real-World Evidence (RWE), which will be increasingly used in our daily life [1].

In the past century, laboratory medicine specialists initiated the automation of analytical techniques to improve laboratory performance and reproducibility [2]. Since then, new features have been progressively incorporated to include magnitudes such as demographic data, chemical kinetics, red flags, fluorescence, and calculations, to name a few. The growing complexity of these techniques has required laboratory professionals to engage in special training and closely collaborate with computer scientists at their workplace. Additionally, laboratory professionals are increasingly requesting the *in vitro* diagnostics industry to integrate new technologies in their analyzers. These synergies will induce a dramatic transformation of healthcare systems towards a truly personalized precision medicine.

Data management tools were integrated into the clinical laboratory via commercially-available laboratory information systems, which were scarcely customizable. Since then, other open-source solutions have emerged to incorporate continuously enhanced capabilities. These enhanced information management systems are tailored to meet specific laboratory needs. Our readers will certainly be familiar with programming languages such as Python, R or SQL, which are increasingly used by professionals for descriptive statistics, command line development or data modeling [3].

The Spanish Society of Laboratory Medicine (SEQC^ML^) has promoted the creation of a new Data Science Working group with the following goals:

– Organizing training activities and collaborating with other professionals for the integration of data science in clinical laboratories.
– Promoting best practices to reduce potential biases and errors in the implementation of new technologies, with the support of the *in vitro* diagnostics industry.
– Ensuring data safety and traceability by establishing a legal and ethical framework.

This Working group is composed of a panel of experts in databases, standardized communication and integration, information systems, programming, and data modeling, among others. Some of the components of this Working Group are also members of national and international associations committed to promoting the use of data science, including the Spanish Federation of Medical Research Associations (FACME) or the European Federation of Clinical Chemistry and Laboratory Medicine (EFLM), and have become a reference for their colleagues.

Other medical societies, such as the Spanish Society of Nephrology and the Spanish Society of Internal Medicine have created working groups similar to the SEQC-ML Data Science Working Group. These groups are contributing to progress in the implementation of artificial intelligence and big data/data mining in their respective fields. These technologies are flexible but complex, thereby requiring the integration of computer engineers or mathematicians in their groups.

These professionals will take an active role in the validation of models, the integration of new technologies into real practice, and the development of a legal and ethical framework. All these actions will ultimately improve sample management processes and test result reporting. The application of new technologies to the clinical laboratory does not represent a threat to human work, but it gives a more relevant role to laboratory professionals, as it requires strong analytical skills and creativity [4].

However, this adaptation process is not exempt from some challenges. Healthcare center managers should direct digitation and help desk efforts towards the application of new techniques that require medical knowledge and provide added value. This way, laboratory specialists interested in new technologies will contribute to advancing in the implementation of data science by applying their knowledge in laboratory medicine for an improved healthcare.
